# Human Resources for eye health in South Asia

**Published:** 2018-07-31

**Authors:** Yuddha Dhoj Sapkota

**Affiliations:** 1 South East Asia Regional Coordinator: International Agency for the Prevention of Blindness, Kathmandu, Nepal.

**Human resources in the right numbers, mix and distribution are key to ensuring universal eye health. It is imperative to get a good sense of the current status and challenges in the different countries in South Asia.**

**Figure:**
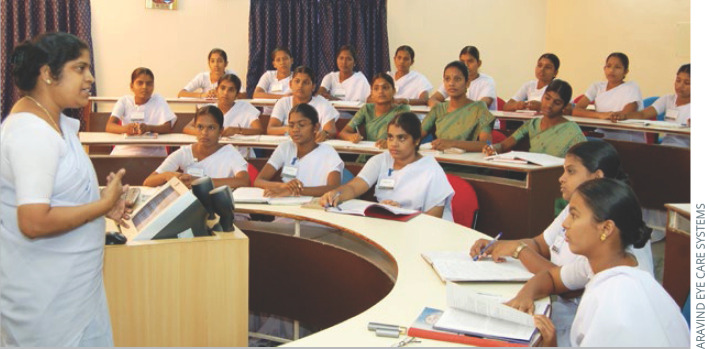
Mid-level Ophthalmic Personnel Training at Aravind Eye Care Systems, Chennai. INDIA

The South Asian region has the largest share of the visual impairment burden in the world. While it has offered many innovative models and solutions that has revolutionised eye care delivery around the world, the region is challenged by a lack of adequate human resources in every cadre of eye care. This article, and this issue of the Community Eye Health Journal South Asia edition, will look at the many contours of this issue and discuss some of the solutions on offer.

The World Health Organization (WHO)'s Global Action Plan 2014-19 (and indeed, all the documents from the original VISION 2020 Initiative in 1999) emphasise the need for skilled human resources for a sustainable and stable eye health delivery system. South Asia is one of the most populous regions of the world and all the countries in the region have varied health systems, governance structures as well as health indicators. However, the good news is that national governments have robust national eye health plans that are also funded.

The International Agency for the Prevention of Blindness's (IAPB) Vision Atlas presents the latest available data on blindness prevalence across regions. We now know that more than 87% of visual impairment in the South Asian region is avoidable. 73 million people live with visual impairment in South Asia. Uncorrected refractive errors (URE) and cataract continue to be the main causes. While the prevalence of visual impairment has come down from 8.47% to 5.74%, there is a clear danger that with an increasing and ageing population, and the epidemics of myopia and diabetic retinopathy these fragile gains will be lost.^1^

## The paradox

The South Asian region is full of innovation, massive public-private collaborations and immense world-changing success stories. Key developments in the region like mass manufacturing of high quality and yet affordable intraocular lenses (IOLs), the pyramid model of eye care delivery, innovations in surgical techniques and the presence of world-class eye hospital networks have been the engines of success here and around the world.

So, how can the two seemingly contradictory facts co-exist in the same region? The inadequacy of eye health resources is a crucial factor in explaining this gap.

Before we explore this in more detail, we must note that the region has successful models of task-sharing with other eye care personnel. The region has many reputed training institutes, with a network of training centres catering to subspecialty training for ophthalmologists and to an extent for optometrists and allied ophthalmic personnel (AOP).

## Current scenario

The three main cadres of human resources in an eye care service delivery system are ophthalmologists, optometrists and AOPs (ophthalmic assistant, ophthalmic technician, ophthalmic nurses and opticians—there is considerable variation in role definition and no internationally accepted understanding of what makes up this cadre exists). The IAPB Vision Atlas provides data on world-wide availability of human resources. While the data has different confidence intervals for different cadres, it gives a macro picture of the status of human resources for eye care in the South Asian region.

**Figure 1:**
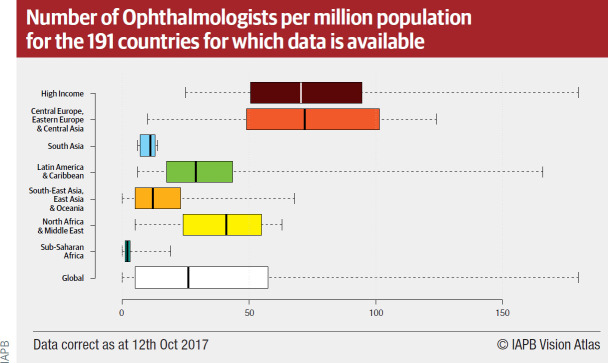
Graph to show the number of ophthalmologists per million population for the 191 countries

Figure 1 and Table 1, show the general inequity in the availability of ophthalmologists across world regions, for example, it highlights the severe resource challenge in South Asia--second only to Sub-Saharan Africa.

The major issues effecting human resources in the South Asian region are:

- Inadequate numbers
- Varying skill levels
- Uneven distribution and
- Low productivity

India, with the largest population of 1.2 billion, also has the largest number of ophthalmologists (20,000). India also has a large training capacity and supports subspeciality training for neighbouring countries as well.

There are inequities in the availability of eye care cadre in rural vs urban areas, especially the distribution of ophthalmologists. This exacerbates the lack of access to eye care. In some countries like Bangladesh and Sri Lanka, allied ophthalmic personnel such as optometrists and ophthalmic assistants are not fully recognised nor accredited to carry out eye care services independently. This means the precious time of ophthalmologists is spent on primary eye care and routine skill-based activities like refraction services. Needless to say, this also effects the overall productivity of ophthalmologists in the country and in the region. Further the lack of surgical training and diagnostic skills during primary ophthalmic training for optometrists or para-ophthalmic assistants necessitates several years of retraining. The annual output of cataract surgeries per ophthalmologists in Nepal is much higher than its neighbouring countries. In Nepal all primary eye care and refraction services are performed by either optometrists or ophthalmic assistants. All countries except Maldives has training capacity for all cadres of eye health human resources. Given their very small population, Bhutan and Maldives do not have sub specialty fellowship training nor such services.

**Table 1:** Available information on the number of eye care personnel in various cadres and the rate per million population.

S. No.	Country	Population	HR Cadre	Number	Ratio per million	Comments
1	Bangladesh	26,00,00,000	Ophthalmologist	1100	4	
			Optometrist	200	0.7	
			AOP	1000	4	
2	Bhutan	7,50,000	Ophthalmologist	8	10	
			Optometrist	4	5	
			AOP	54	72	
3	India	1,20,00,00,000	Ophthalmologist	20,000	16	
			Optometrist	9,000	7	
			AOP	40,000	33	
4	Nepal	3,00,00,000	Ophthalmologist	308	10	
			Optometrist	470	15	
			AOP	950	31	
**5**	Pakistan	2,00, 000, 000	Ophthalmologist	2590	13	
			Optometrist	1605	8	
			AOP	2156	11	
**6**	Maldives	3,50,000	Ophthalmologist	10	28	(8 are expat)
			Optometrist	10	28	(All expat)
**7**	Sri Lanka	2,10,00,000	Ophthalmologist	195	9	

## Conclusion

The solutions to addressing this inadequacy in human resources in the countries of the region are diverse. Adoption of strategies like competency based assessments (CBA) and skill certification are emerging as innovative ways to fast-track the development of allied ophthalmic personnel and ensure that they are competent and reliable. Multiple creative—and successful—approaches are being deployed to bring and retain AOP in rural settings across the region. This issue presents a number of these examples from the region. In conclusion we need every effort and innovation to develop and retain skilled human resources, so that we can ensure that eye health reaches everyone, everywhere.

**Figure:**
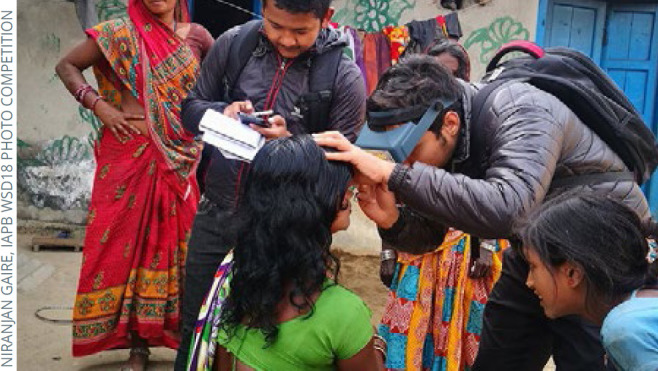
A trachoma elimination survey in Nepal. NEPAL
